# Improving duplicated nodes position in vertebrate gene trees

**DOI:** 10.1186/1471-2105-16-S3-A9

**Published:** 2015-02-13

**Authors:** Amélie Peres, Hugues Roest Crollius

**Affiliations:** 1 Ecole Normale Supérieure, Institut de Biologie de l'ENS, IBENS, France

## Background

While gene phylogenies are essential for many biological evolutionary studies, phylogenetic reconstructions are difficult to model, especially when they include gene duplications. In this study, we have developed a method to improve the positions of duplications in gene trees produced by TreeBest, a widely used method at the core of the "Ensembl compara" pipeline[1].

## Results

In order to automatically identify incorrectly positioned duplications, we investigated a method that relies on the confidence score, a measure between 0 and 1 introduced by TreeBest that is assigned to each duplication node. This score reflects the ratio between the number of species with a duplicated gene and the total number of species derived from this node. A well-supported duplication will thus have a score closer to 1.

With our method, if a duplication node is considered to be poorly supported it is replaced by a speciation node, and the duplication is moved to the following node which is tested using the same method. If the new duplication node passes the test, the duplication is maintained at this new position in the tree.

To test our method comprehensively, we ran it on all 20194 phylogenetic trees available in the Ensembl compara database version 71. The resulting 20194 new edited gene trees were then compared with the original Ensembl gene trees by feeding both databases to AGORA[2], an algorithm developed in our laboratory to reconstruct ancestral gene orders. This tool allowed us to assess the quality of the new gene trees as its performances are very sensitive to the quality of the input gene trees, in particular because the length of the reconstructed ancestral chromosomal regions varies substantially depending on the quality of the input gene trees.

**Figure 1:**
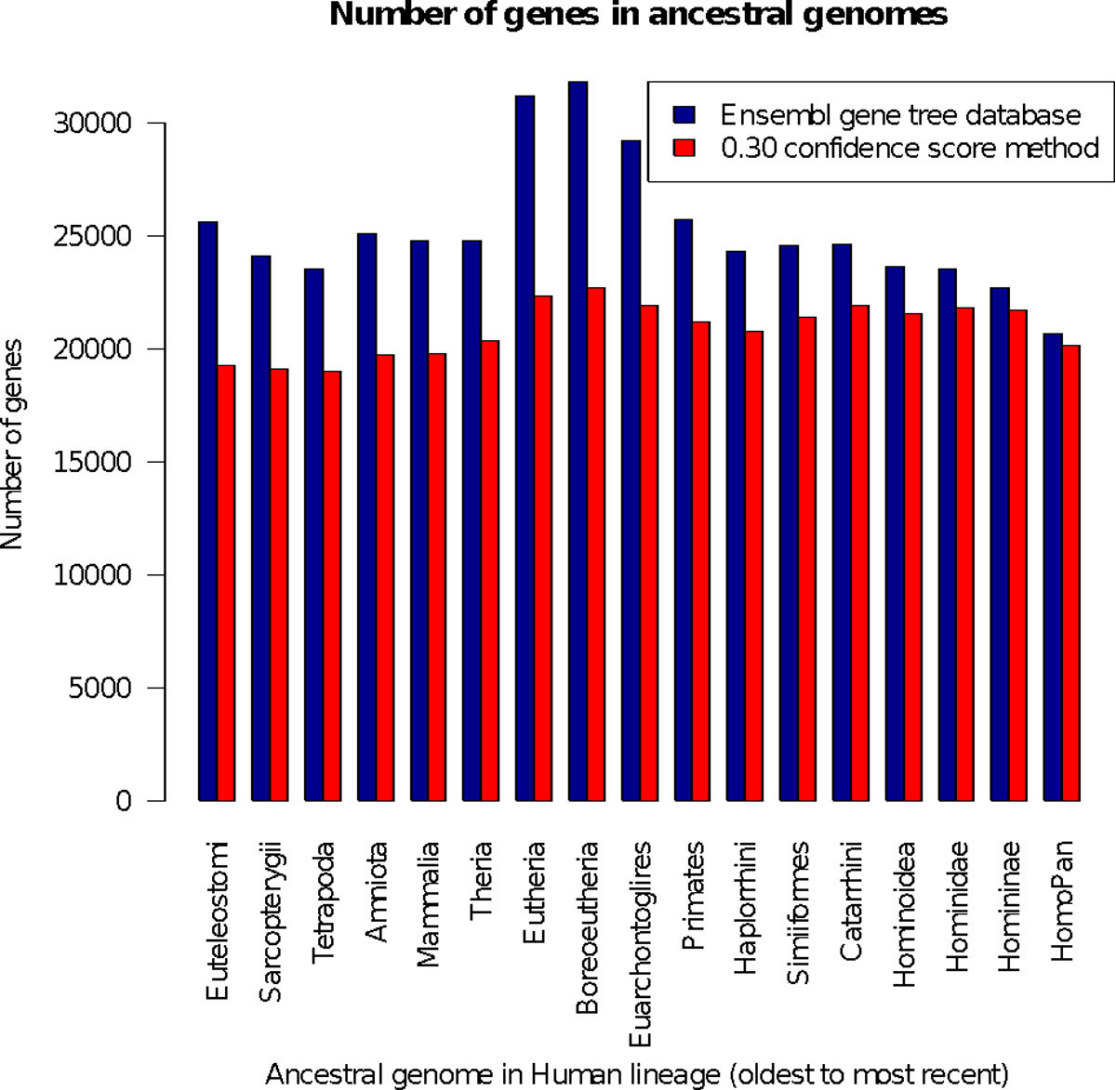
**Number of genes in ancestral genomes obtained with the original Ensembl gene trees database (in blue) and with edited gene trees with the confidence score method and a threshold of 0.3 (in red)**.

With the Ensembl gene trees, the number of ancestral genes increases and decreases rapidly during time, whereas with edited gene trees, the number of genes is more constant (Figure 1), which is more likely from an evolutionary perspective. Additionally, in some cases the number of ancestral genes is more reasonable. Such is the case for the common ancestor for primates and rodents, Boreoeutheria, where its genome reconstruction with the Ensembl gene trees has 30 000 genes, but its genome reconstructed with our edited gene trees is only 20 000 genes large. The latter value is much closer to what one would expect because all modern Boreoeutheria descendant genomes contain between 20 000 and 25 000 genes.

**Figure 2:**
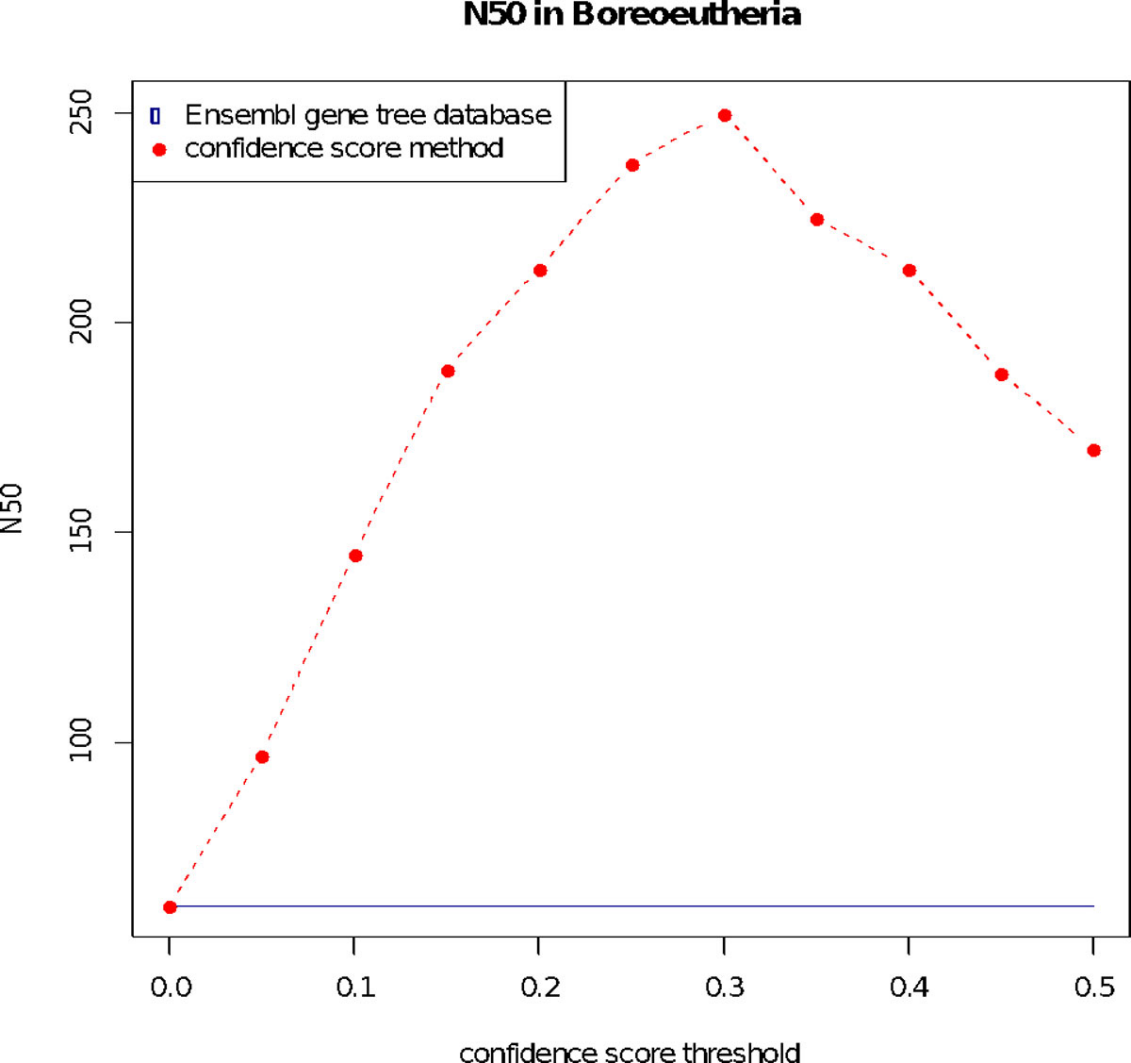
**N50 measurement for the Boreoeutheria genome reconstruction with the original Ensembl gene trees database (in blue) and with our edited gene trees with the confidence score method (in red)**. Edited trees significantly improve the N50. The optimal threshold is 0.3. Results are similar for all other ancestral genomes.

We also test the N50 measurement, which is the size of an ancestral block such as 50% of genes are in larger blocks, for all reconstructed ancestral genomes. A higher N50 indicates a better ancestral genome reconstruction. Edited gene trees using our confidence score method significantly improve the N50 and most notably with a threshold of 0.3 that was obtained empirically (Figure 2).

## Conclusions

We find that using the confidence score method significantly improves the positions of duplications within gene trees when compared to the initial Ensembl gene tree database. The optimal value is obtained with a threshold score of 0.3, at which 39% of the 197 894 duplication nodes of the Ensembl gene tree database are edited, resulting in an increase in the N50 length for the ancestral reconstruction of the 58 vertebrate ancestors. These results suggest that our improved gene trees are more reliable.
